# Discovery of a highly potent and selective Bruton’s tyrosine kinase inhibitor avoiding impairment of ADCC effects for B-cell non-Hodgkin lymphoma

**DOI:** 10.1038/s41392-020-00309-1

**Published:** 2020-09-14

**Authors:** Juan Liu, Qianmao Liang, Aoli Wang, Fengming Zou, Ziping Qi, Kailin Yu, Qingwang Liu, Cheng Chen, Jing Liu, Qingsong Liu

**Affiliations:** 1 Anhui Province Key Laboratory of Medical Physics and Technology, Institute of Health and Medical Technology, Hefei Institutes of Physical Science, Chinese Academy of Sciences, 230031 Hefei, Anhui P. R. China; 2 University of Science and Technology of China, 230036 Hefei, Anhui P. R. China; 3 Hefei Cancer Hospital, Chinese Academy of Sciences, 230031 Hefei, Anhui P. R. China; 4 Precision Medicine Research Laboratory of Anhui Province, 230088 Hefei, Anhui P. R. China; 5 Precision Targeted Therapy Discovery Center, Institute of Technology Innovation, Hefei Institutes of Physical Science, Chinese Academy of Sciences, 230088 Hefei, Anhui P. R. China; 6 Institutes of Physical Science and Information Technology, Anhui University, 230601 Hefei, Anhui P. R. China

**Keywords:** Haematological cancer, Drug screening, Haematological cancer, Drug screening

**Dear Editor**,

Bruton’s tyrosine kinase (BTK) plays a crucial role in the B-cell receptor (BCR) signaling which is essential for B-cell proliferation, differentiation, and cell migration. Aberrant BCR activation has been identified as a major pathogenic factor in several B-cell non-Hodgkin lymphoma (B-NHL) subtypes, including diffuse large B-cell lymphoma (DLBCL), mantle cell lymphoma (MCL), follicular lymphoma (FL), and chronic lymphocytic leukemia (CLL).^1^ Therefore, BTK has been recognized as a validated therapeutic target for B-cell malignancies. Ibrutinib, the first approved BTK inhibitor that binds irreversibly to cysteine residue 481, has shown potent clinical activity in the majority of CD20 positive B-cell malignancies.^2^ However, due to the inhibition of off-target kinases such as EGFR, ITK, and TXK, which have a cysteine residue at the identical position of Cys481 of BTK, Ibrutinib also results in some adverse events, such as the antagonizing Rituximab-dependent NK-cell-mediated antibody-dependent cell-mediated cytotoxicity (ADCC) due to its irreversible binding to ITK, which is required for FcR-stimulated NK cell function.^3^ Although several secondary generation inhibitors have shown improved selectivity,^4,5^ more pharmacologically diverse novel inhibitors are still highly demanded in the clinic.

Here, we report the discovery of a novel covalent BTK inhibitor, CHMFL-BTK-85 (abbreviated as compd. 85) (chemical structure shown in Fig. 1a), which achieves high potency against BTK and selectivity over other protein kinases. The ADP-Glo^TM^ biochemical assay with purified BTK protein showed that compd. 85 exhibited an IC_50_ value of 11.5 nM against BTK which was over 15-fold more potent than the reversible version compound CHMFL-BTK-85R (chemical structure shown in Supplementary Fig. S1 and Fig. 1b), indicating a covalent binding mode. Immunoblotting analysis of the autophosphorylation at Y223 site of BTK wt and C481S mutant in the transiently transfected HEK293 cells also proved that the covalent binding was via the Cysteine 481 residue (Fig. 1c). To further confirm that compd. 85 could covalently bind to wide-type native BTK in cell, we then conducted a target-engagement assay using the biotinylated analog of compd. 85, i.e., CHMFL-BTK-85B (Supplementary Fig. S2), in REC-1 cell which expresses native BTK kinase. The data showed that after 2 h, compd. 85 could dose-dependently compete with CHMFL-BTK-85B and at 1 μM concentration it could almost completely covalently label all of the available BTK (Supplementary Fig. S3). The ADP-Glo^TM^ biochemical assay with kinases targeted by Ibrutinib showed that compd. 85 only exhibited moderate selectivity over BMX (10-fold), but it achieved more than 1000-fold selectivity over all of the others including ITK, BLK, EGFR, HER2, HER4, JAK3, and TXK kinases (Fig. 1d and Supplementary Table S1). To further investigate the selectivity of compd. 85 in the kinome, we then tested it among 468 kinases/mutants with KINOMEscan^TM^ technology. The results showed that it was highly selective (*S* Score (1) = 0.00) at the concentration of 1 μM and only BTK kinase was revealed as the strong binding target (Fig. 1e and Supplementary Table S2).

**Fig. 1:**
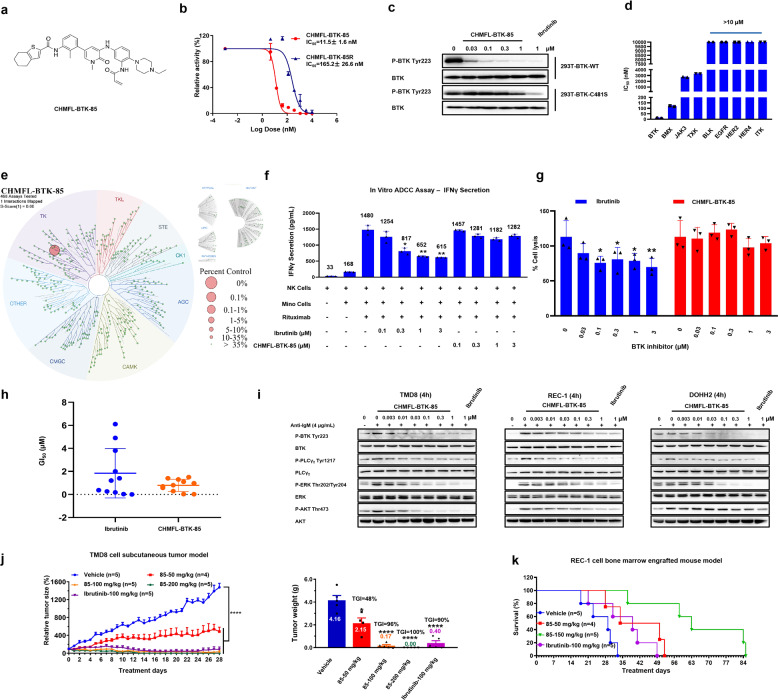
CHMFL-BTK-85 could avoid impairment of the ADCC effects in B-cell non-Hodgkin lymphoma. **a** Chemical structure of CHMFL-BTK-85. **b** ADP-Glo^TM^ assay determination of the IC_50_ values of CHMFL-BTK-85 and CHMFL-BTK-85R against BTK kinase. **c** CHMFL-BTK-85 inhibited the cellular autophosphorylation Tyr223 of WT BTK and the C481S mutant in transiently transfected HEK293 cells. The blots were cropped for improved clarity and conciseness. **d** ADP-Glo^TM^ assay determination of the IC_50_ values of CHMFL-BTK-85 against BTK, BLK, BMX, EGFR, HER2, HER4, ITK, JAK3, and TXK kinases. **e** Kinome-wide selectivity profiling of CHMFL-BTK-85 with DiscoverX’s KINOMEscan^TM^ technology (http://www.kinomescan.com). The red circles indicated kinases bound, and circle size indicated relative binding affinity compared to DMSO (Ctrl%). The complete dataset was shown in Supplementary Table 2. **f** Mino cells and NK cells were co-seeded and treated with vehicle or various concentrations of BTK inhibitors in the presence of Rituximab; interferon (IFN)-γ levels in the conditioned medium were measured as a readout of the assay. **g** Mino cells and NK cells were co-seeded and treated with vehicle or various concentrations of Ibrutinib and CHMFL-BTK-85 in the presence of Rituximab. Cytotoxicity of the target cells was determined by lactate dehydrogenase release into the culture medium. **h** Anti-proliferative effects of CHMFL-BTK-85 against a panel of B-cell lymphoma cell lines. The cells were treated with Ibrutinib and CHMFL-BTK-85 (maximum concentration 10 μM) for 72 h, and then cell viability was measured using the CellTiter–Glo assay (Error bars, mean ± SEM, *n* = 3). **i** The phosphorylation levels of BTK (Tyr223), PLCγ_2_ (Tyr1217), ERK1/2 (Thr202/Tyr204), and AKT (Ser473) were detected by western blot in TMD8, REC-1, and DOHH2 cell lines. These cells were incubated with the indicated concentrations of CHMFL-BTK-85 for 4 h before lysis. **j** Anti-tumor effects of CHMFL-BTK-85 with once daily (QD) dosing at 50, 100, and 200 mg/kg. The total study length was 28 days. (Left) Representative graphs of relative tumor size are shown. (Right) Representative graphs of tumor weight of different groups are shown. **k** Anti-tumor efficacy of CHMFL-BTK-85 in the bone marrow engrafted mouse model. Kaplan–Meier plots of survival. The disseminated NOD/SCID mice were intravenously inoculated with REC-1 cells and received daily oral administration of CHMFL-BTK-85 dosing at 50 and 150 mg/kg. The total study length was 84 days, and each treatment group contained 4–5 animals. Date are shown as mean ± SEM, *P-value < 0.05, **P-value < 0.01, ***P-value < 0.001, and ****P < 0.0001

Given the fact that Ibrutinib could impair anti-CD20 antibodies exerted antibody drug-dependent NK-cell-mediated cytotoxicity (ADCC) due to the selectivity problem, we then examined compd. 85 in the human NK cells co-cultured with the Mino cells or SK-OV-3 cells in the presence of Rituximab or Herceptin. Ibrutinib strongly inhibited Rituximab and Herceptin-induced IFN-γ secretion in the NK cells in a dose-dependent manner between 0.1 and 3 μM, meanwhile compd. 85 showed no apparent inhibition up to 3 μM, which recapitulated its weak ITK inhibitory activity (Fig. 1f and Supplementary Fig. S4). Furthermore, Ibrutinib significantly impaired the antibody-dependent NK-cell-mediated cytotoxicity (ADCC) against Mino and SK-OV-3 cells in the in vitro lactate dehydrogenase (LDH) release experiment (Fig. 1g and Supplementary Fig. S5). In comparison, compd. 85 did not affect the killing efficacy of NK cells which further confirmed that it would not abrogate the ADCC effect.

We next evaluated the anti-proliferative effects of compd. 85 against a panel of B-cell lymphoma cell lines. Overall, it was potent to all these cell lines (GI_50_s: <2 μM) while Ibrutinib exhibited a relatively random trend (Fig. 1h and Supplementary Table S3). In addition, it displayed similar potency to the second generation BTK kinase inhibitor Acalabrutinib in TMD8 (DLBCL) and REC-1 (MCL) cells. In TMD8, REC-1, and DOHH2 cells, compd. 85 potently blocked the BTK Y223 autophosphorylation (<10 nM) and inhibited the phosphorylation of downstream mediators such as PLCγ2, ERK, AKT (Fig. 1i), and p-NF-κB p65 (Supplementary Fig. S6). In addition, dose-dependent apoptotic induction and cell cycle arrest were observed in these cell lines (Supplementary Fig. S7a, b).

The in vivo pharmacokinetic study showed that compd. 85 bore acceptable bioavailability (*F* = 29%) and suitable half-life (*T*_1/2_ = 2.9 h), and good drug exposure (AUC_0−t_ = 2145 ng/mL) for oral administration at 10 mg/kg in rats (Table S4). The dose escalation study showed that compd. 85 was well tolerated up to 800 mg/kg/day dosage for continuous 14 days with no apparent toxicity observed (Supplementary Fig. S8a, b). In addition, compd. 85 exhibited dose-dependent anti-tumor efficacy in the TMD8 cell (DLBCL)-inoculated xenograft mouse model and the tumor growth inhibition (TGI) of 96% was achieved at 100 mg/kg/day dosage, which was better than Ibrutinib (TGI = 90%) at the same dosage (Fig. 1j). Again, no weight loss or any other obvious signs of toxicity were observed (Supplementary Fig. S9a). In the TMD8 tumor tissues, the BTK-mediated signaling was dose-dependently inhibited by compd. 85, which was consistent with its in vivo anti-tumor phenotype and confirmed its on-target effect (Supplementary Fig. S9b). In order to further evaluate the in vivo efficacy of compd. 85, we then examined it in the REC-1 cell (MCL)-inoculated xenograft mouse model, 100 mg/kg/day dosage of compd. 85 slowed down the tumor progression and showed a TGI of 65% without obvious signs of toxicity, which was slightly better than Ibrutinib (TGI = 59%) and Acalabrutinib (TGI = 58%) at the same dosage (Supplementary Fig. S10a, b). In total, 150 mg/kg/day dosage of compd. 85 could achieve TGI of 79%. Evaluation of drug enrichment in the tumor showed that at the same dosage (100 mg/kg) compd. 85 could reach a much higher concentration (2.37 μM) relative to Ibrutinib (1.23 μM) and Acalabrutinib (1.30 μM). This better in tumor PK profile may partially explain the better in vivo efficacy of compd. 85. In the REC-1 cell-mediated orthogonal mouse model of bone marrow engraftment, compd. 85 dose-dependently extended the median survival time of mice to 42 days at 50 mg/kg/day dosage and meanwhile exhibited better efficacy than Ibrutinib (median survival time was 39 days at 100 mg/kg/day dosage). At 150 mg/kg/day dosage, compd. 85 could even extend the median survival time of mice to 63 days (Fig. 1k and Supplementary Fig. S10c).

In short, we have discovered a novel highly selective covalent BTK kinase inhibitor CHMFL-BTK-85, which did not affect the NK-cell-mediated ADCC effects and showed good in vitro and in vivo anti-tumor efficacies. These data support further investigation of CHMFL-BTK-85 as a potential clinical drug candidate, especially in combination with anti-CD20 antibodies, for B-cell non-Hodgkin lymphoma.

## Supplementary information

Supplementary Information

Supplementary Table S2

## Data Availability

The datasets used and/or analyzed to support the findings of this study are available in this paper or the Supplementary Information. Any other raw data that support the findings of this study are available from the corresponding author upon reasonable request.

## References

[1] Pal Singh, S., Dammeijer, F. & Hendriks, R. W. Role of Bruton’s tyrosine kinase in B cells and malignancies. *Mol. Cancer*. 10.1186/s12943-018-0779-z (2018). 10.1186/s12943-018-0779-z PMC5817726 29455639

[2] Mathur, R. Burton’s Tyrosine Kinase Inhibition by Ibrutinib: current status. *J. Leuk*. 10.4172/2329-6917.1000e113 (2015).

[3] Kohrt HE (2014). Ibrutinib antagonizes rituximab-dependent NK cell-mediated cytotoxicity. Blood.

[4] Byrd JC (2016). Acalabrutinib (ACP-196) in relapsed chronic lymphocytic leukemia. New Engl. J. Med..

[5] Guo Y (2019). Discovery of zanubrutinib (BGB-3111), a novel, potent, and selective covalent inhibitor of Bruton’s tyrosine kinase. J. Med. Chem..

