# Supplementary material for: Discovery of a highly potent and selective Bruton’s tyrosine kinase inhibitor avoiding impairment of ADCC effects for B-cell non-Hodgkin lymphoma
Source: Signal Transduct Target Ther. 2020 Sep 14;5:200. doi: 10.1038/s41392-020-00309-1 (PMC7490411; doi:10.1038/s41392-020-00309-1)
Supplement: Supplementary file 1 — Supplementary Information [file 41392_2020_309_MOESM1_ESM.docx]

Supplementary Materials for

**Discovery of a highly potent and selective Bruton’s tyrosine kinase inhibitor avoiding impairment of ADCC effects for** **B-cell non-Hodgkin lymphoma**

Juan Liu^1,2,7^, Qianmao Liang^1,2,7^, Aoli Wang^1,3,7^, Fengming Zou^1,3,7^, Ziping Qi^1,3^, Kailin Yu^1^, Qingwang Liu^1,3,4,5^, Cheng Chen^1,2,3^, Jing Liu^1,2,3*^, Qingsong Liu^1,2,3,4,5,6*^

1. Anhui Province Key Laboratory of Medical Physics and Technology, Institute of Health and Medical Technology, Hefei Institutes of Physical Science, Chinese Academy of Sciences, Hefei, Anhui 230031, P. R. China
2. University of Science and Technology of China, Hefei, Anhui 230036, P. R. China
3. Hefei Cancer Hospital, Chinese Academy of Sciences, Hefei, Anhui 230031, P. R. China
4. Precision Medicine Research Laboratory of Anhui Province, Hefei, Anhui 230088, P. R. China
5. Precision Targeted Therapy Discovery Center, Institute of Technology Innovation, Hefei Institutes of Physical Science, Chinese Academy of Sciences, Hefei, Anhui 230088, P. R. China
6. Institutes of Physical Science and Information Technology, Anhui University, Hefei, Anhui 230601, P. R. China
7. These authors contribute equally

**Corresponding Authors**

* E-mail: [qsliu97@hmfl.ac.cn](mailto:qsliu97@hmfl.ac.cn). Phone: 86-551-65596201 (Q.L.)

* E-mail: [jingliu@hmfl.ac.cn](mailto:jingliu@hmfl.ac.cn). Phone: 86-551-65593186 (J.L.).

**This PDF file includes:**

Materials and Methods

Figures. S1 to S10

Tables S1 to S4

**Material and Methods**

**Chemicals**

Ibrutinib, Acalabrutinib, Rituximab, and Herceptin were purchased from MedChemexpress (Shanghai, P.R.China).

**Synthesis of CHMFL-BTK-85**

General methods. All reagents and solvents were purchased from commercial sources and used as obtained. ^1^H NMR and ^13^C NMR spectra were recorded with a Bruker 500 NMR spectrometer and referenced to deuterium dimethyl sulfoxide (DMSO-*d_6_*). Chemical shifts were expressed in ppm. In the NMR tabulation, s indicates singlet; d, doublet; t, triplet; q, quartet; m, multiplet; and br, broad peak. Mass spectra was measured with Agilent 6224 TOF using an ESI source coupled to an Agilent 1260 Infinity HPLC system operating in reverse mode with an Agilent Eclipse Plus C18 1.8μm 3.0 X 50 mm column. Flash column chromatography was conducted using silica gel (SiliCycle 40−64 μm). The purity of all compounds were above 95% purity as determined by an Agilent 1260 Infinity HPLC with UV detection at 254 nm.

4-(4-ethylpiperazin-1-yl)-3-nitroaniline (**S1**). To a solution of 4-fluoro-3-nitroaniline (6.24 g, 40.0mmol, 1.00 equiv.) in DMF (50mL) was added 1-ethylpiperazine (6.84 g, 60.0 mmol, 1.50 equiv.) and K_2_CO_3_ (11.0 g, 80.0mmol, 2.00 equiv.) at room temperature. The reaction mixture was heated to 80 ^o^C for 8h. The resulting mixture was poured into water (500mL). The mixture was filtered to give **S1**(7.90 g, yield =79%) as a red solid. ^1^H NMR (500 MHz, DMSO) δ 7.16 (d, *J* = 8.7 Hz, 1H), 6.86 (d, *J* = 2.6 Hz, 1H), 6.79 (dd, *J* = 8.7, 2.6 Hz, 1H), 5.43 (s, 2H), 2.81 (t, *J* = 4.6 Hz, 4H), 2.41 (s, 4H), 2.34 (q, *J* = 7.2 Hz, 2H), 1.00 (t, *J* = 7.2 Hz, 3H). ^13^C NMR (126 MHz, DMSO) δ 147.49, 146.26, 134.63, 124.41, 118.71, 108.03, 53.23, 53.19, 52.09, 12.45. LC-MS(ESI, m/z): 251.2[M+H]^+^.

5-bromo-3-((4-(4-ethylpiperazin-1-yl)-3-nitrophenyl)amino)-1-methylpyridin-2(1*H*)-one (**S2**). To a solution of **S1** (2.34 g, 9.36 mmol, 1.00 equiv) in dioxane (15 mL) was added 3,5-dibromo-1-methylpyridin-2(1*H*)-one (2.50 g, 9.36mmol, 1.00 equiv), Pd_2_(dba)_3_ (0.43g, 0.47 mmol, 0.05equiv), Xantphos (0.38g, 0.66 mmol, 0.07equiv) and Cs_2_CO_3_ (9.15g, 28.08mmol, 3.00 equiv.) at room temperature in seal tube under argon atmosphere. The reaction mixture was stirred at 120^o^C for 12h. The solvent was then removed under reduced pressure and the residue was diluted with water and extracted with dichloromethane. The combined organic layers were washed with water, brine and dried over anhydrous Na_2_SO_4_. Evaporation of the solvent provided the crude product, which was purified by flash chromatography with MeOH in DCM 0-7% to give **S2**(2.65g, yield =65%) as a red solid. ^1^H NMR (500 MHz, DMSO) δ 8.22 (s, 1H), 7.68 (d, *J* = 2.6 Hz, 1H), 7.51 (dd, *J* = 8.9, 2.6 Hz, 1H), 7.46 (d, *J* = 2.3 Hz, 1H), 7.33 (d, *J* = 8.9 Hz, 1H), 6.96 (d, *J* = 2.3 Hz, 1H), 3.50 (s, 3H), 2.97 – 2.89 (m, 4H), 2.46 (s, 4H), 2.36 (q, *J* = 7.1 Hz, 2H), 1.01 (t, *J* = 7.2 Hz, 3H). ^13^C NMR (126 MHz, DMSO) δ 156.67, 145.01, 140.14, 136.48, 134.64, 127.56, 125.33, 123.40, 116.24, 113.22, 97.83, 52.90, 52.30, 52.04, 37.47, 12.43. LC-MS(ESI, m/z): 436.1[M+H]^+^.

2-methyl-3-(4,4,5,5-tetramethyl-1,3,2-dioxaborolan-2-yl)aniline(**S3**). To a solution of 3-bromo-2-methylaniline (3.72 g, 20mmol, 1.00 equiv) in dioxane (100 mL) was added bis(-pinacolato)diboron (7.62g, 30 mmol, 1.50 equiv), Pd(dppf)Cl_2_•DCM(0.82 g, 1mmol) and KOAc (5.88 g, 60mmol). The reaction mixture was stirred at 90 ^o^C for 8 h under argonatmosphere, and then concentrated to remove the dioxane. The residue was diluted with water (500 mL) and extracted with DCM (3 * 200 mL). The combined organic layers were washed with water, brine and dried over anhydrous Na_2_SO_4_. Evaporation of the solvent provided the crude product, which was purified by flash chromatography with0~5% EA in pe to afford **15-22** (4.23 g, yield =90%) as a white solid. ^1^H NMR (500 MHz, DMSO) δ 6.87 (d, *J* = 4.3 Hz, 2H), 6.71 (t, *J* = 4.6 Hz, 1H), 4.72 (s, 2H), 2.22 (s, 3H), 1.28 (s, 12H). ^13^C NMR (126 MHz, DMSO) δ 146.88, 127.39, 125.81, 124.13, 117.16, 83.44, 25.29, 25.10, 16.32. LC-MS(ESI, m/z): 234.2[M+H]^+^.

*N*-(2-methyl-3-(4,4,5,5-tetramethyl-1,3,2-dioxaborolan-2-yl)phenyl)-4,5,6,7-tetrahydrobenzo [*b*]thiophene-2-carboxamide (**S4**).To a solution of **S3** (2.33 g, 10mmol) in DMF (80 mL) was added 4,5,6,7-tetrahydrobenzo[*b*]thiophene-2-carboxylic acid (1.82g, 10 mmol), HATU (4.56 g, 12mmol) and DIPEA (2.58 g, 20 mmol). The reaction mixture was stirred at RT for 4 h. The solution was slowly poured into water and stirred for 10 mins. A large amount of white solid precipitated and filtered to give a crude product **S4**（3.81 g,yield = 96%）as a white solid. ^1^H NMR (500 MHz, DMSO) δ 9.73 (s, 1H), 7.64 (s, 1H), 7.54 (d, *J* = 7.4 Hz, 1H), 7.35 (d, *J* = 7.4 Hz, 1H), 7.20 (t, *J* = 7.6 Hz, 1H), 2.75 (t, *J* = 5.5 Hz, 2H), 2.61 (t, *J* = 5.6 Hz, 2H), 2.36 (s, 3H), 1.84 – 1.69 (m, 4H), 1.31 (s, 12H). LC-MS(ESI, m/z): 398.2[M+H]^+^.

*N*-(3-(5-((4-(4-ethylpiperazin-1-yl)-3-nitrophenyl)amino)-1-methyl-6-oxo-1,6-dihydropyridin-3-yl)-2-methylphenyl)-4,5,6,7-tetrahydrobenzo[*b*]thiophene-2-carboxamide (**S5**). To a solution of **S2** (2.18g, 5mmol) in dioxane (100 ml) was added **S4** (2.38 g, 6mmol), Pd(PPh_3_)_4_ (0.29 g, 0.25mmol), Na_2_CO_3_ (1.06 g, 10mmol) and H_2_O (5 mL). The reaction mixture was stirred at 100 ^o^C for 12h under argonatmosphere, and then concentrated to remove the dioxane. The residue was diluted with water (100 mL) and extracted with DCM (3 * 80 mL). The combined organic layers were washed with water, brine and dried over anhydrous Na_2_SO_4_. Evaporation of the solvent provided the crude product, which was purified by flash chromatography with 0-7% MeOH in DCM to afford **S5** (2.44 g, yield =78%) as a red solid. ^1^H NMR (500 MHz, DMSO) δ 9.79 (s, 1H), 8.12 (s, 1H), 7.66 (d, *J* = 2.6 Hz, 2H), 7.48 (dd, *J* = 8.9, 2.6 Hz, 1H), 7.30 (dd, *J* = 12.3, 8.1 Hz, 2H), 7.24 (dd, *J* = 13.5, 4.8 Hz, 2H), 7.16 (d, *J* = 7.3 Hz, 1H), 6.96 (d, *J* = 2.0 Hz, 1H), 3.59 (s, 3H), 2.96 – 2.84 (m, 4H), 2.80 – 2.71 (m, 2H), 2.61 (t, *J* = 5.5 Hz, 2H), 2.43 (s, 4H), 2.38 – 2.30 (m, 3H), 2.17 (s, 3H), 1.85 – 1.68 (m, 4H), 1.00 (t, *J* = 7.2 Hz, 3H). LC-MS(ESI, m/z): 626.3[M+H]^+^.

1. (3-(5-((3-acrylamido-4-(4-ethylpiperazin-1-yl)phenyl)amino)-1-methyl-6-oxo-1,6-dihydropyridin-3-yl)-2-methylphenyl)-4,5,6,7-tetrahydrobenzo[*b*]thiophene-2-carboxamide (**CHMFL-BTK-85**). To a solution of **S5** (0.63 g, 1mmol) in MeOH was added Pd/C(10% Pd,55% water) (0.06 g). The reaction mixture was stirred at room temperature for 4 h, and then resulting mixturefiltered through a pad of Celite. The filtrate was concentrated to give a crude *N*-(3-(5-((3-amino-4-(4-ethylpiperazin-1-yl)phenyl)amino)-1-methyl-6-oxo-1,6-dihydropyridin-3-yl)-2-methylphenyl)-4,5,6,7-tetrahydrobenzo[*b*]thiophene-2-carboxamide (0.39 g). To a solution of the *N*-(3-(5-((3-amino-4-(4-ethylpiperazin-1-yl)phenyl)amino)-1-methyl-6-oxo-1,6-dihydropyridin-3-yl)-2-methylphenyl)-4,5,6,7-tetrahydrobenzo[*b*]thiophene-2-carboxamide (0.45 g, 0.75mmol) in dry DCM (20ml) was added acryloyl chloride (65mg, 0.72mmol) and DIPEA (168mg, 1.30mmol) at 0 ^o^C. Then the reaction mixture was stirred at room temperature for 10 min, and then quenched with NaHCO_3_ (aq) and extracted with DCM (3 * 150 mL). The combined organic layers were washed with water, brine and dried over anhydrous Na_2_SO_4_. Evaporation of the solvent provided the crude product, which was purified by flash chromatography with0~9% MeOH in DCM to afford **CHMFL-BTK-85** (351 mg, two steps yield =54%) as a light redsolid. ^1^H NMR (500 MHz, DMSO) δ 9.77 (s, 1H), 9.03 (s, 1H), 8.08 (s, 1H), 7.70 (s, 1H), 7.65 (s, 1H), 7.29 (d, *J* = 7.4 Hz, 1H), 7.23 (t, *J* = 7.6 Hz, 1H), 7.19 (d, *J* = 6.9 Hz, 1H), 7.14 (dd, *J* = 5.1, 3.2 Hz, 2H), 6.99 (dd, *J* = 8.6, 2.2 Hz, 1H), 6.95 (d, *J* = 1.7 Hz, 1H), 6.59 (dd, *J* = 16.9, 10.3 Hz, 1H), 6.26 – 6.17 (m, 1H), 5.72 (d, *J* = 11.0 Hz, 1H), 3.59 (s, 3H), 2.77 – 2.75 (m, 6H), 2.60 (t, *J* = 5.4 Hz, 2H), 2.54 (s, 4H), 2.38 (q, *J* = 7.1 Hz, 2H), 2.19 (s, 3H), 1.83 – 1.70 (m, 4H), 1.02 (t, *J* = 7.2 Hz, 3H). ^13^C NMR (126 MHz, DMSO) δ 163.48, 160.61, 157.15, 141.64, 139.35, 137.88, 137.68, 137.05, 136.39, 135.85, 133.44, 133.38, 132.72, 132.57, 129.97, 128.03, 127.16, 126.63, 126.44, 126.09, 121.29, 119.52, 116.21, 113.10, 112.33, 53.08, 52.19, 52.11, 37.64, 25.47, 25.17, 23.31, 22.68, 16.00, 12.55. LC-MS (ESI, m/z): 650.3[M+H]^+^.

**(CHMFL-BTK-85R)**. To a solution of **S5** (43 mg, 0.069mmol) in MeOH (20ml)was added Pd/C(10% Pd,55% water) (10 mg). The reaction mixture was stirred at room temperature for 4 h, and then resulting mixturefiltered through a pad of Celite. The filtrate was concentrated to give a crude N-(3-(5-((3-amino-4-(4-ethylpiperazin-1-yl)phenyl)amino)-1-methyl-6-oxo-1,6-dihydropyridin-3-yl)-2-methylphenyl)-4,5,6,7-tetrahydrobenzo[b]thiophene-2-carboxamide (35 mg). To a solution of the N-(3-(5-((3-amino-4-(4-ethylpiperazin-1-yl)phenyl)amino)-1-methyl-6-oxo-1,6-dihydropyridin-3-yl)-2-methylphenyl)-4,5,6,7-tetrahydrobenzo[b]thiophene-2-carboxamide (35 g, 0.059mmol) in dry DCM (10ml) was added propionyl chloride (6.5mg, 0.071mmol) and DIPEA (16mg, 0.12mmol) at 0 oC. Then the reaction mixture was stirred at room temperature for 10 min, and then quenched with NaHCO3 (aq) and extracted with DCM (3 * 15 mL). The combined organic layers were washed with water, brine and dried over anhydrous Na2SO4. Evaporation of the solvent provided the crude product, which was purified by flash chromatography with0~9% MeOH in DCM to afford CHMFL-BTK-85R (27 mg, two steps yield =61%) as a light solid. 1H NMR (500 MHz, DMSO) δ 9.78 (s, 1H), 8.75 (s, 1H), 8.06 (s, 1H), 7.67 – 7.65 (m, 2H), 7.27 (d, J = 7.7 Hz, 1H), 7.22 (t, J = 7.6 Hz, 1H), 7.17 (d, J = 6.2 Hz, 1H), 7.14 – 7.11 (m, 2H), 6.95 (d, J = 8.2 Hz, 1H), 6.90 (s, 1H), 3.58 (s, 3H), 2.92 (br, 6H), 2.76 (t, J = 5.7 Hz, 2H), 2.60 (t, J = 5.7 Hz, 2H), 2.51 (s, 2H), 2.45 – 2.37 (m, 2H), 2.16 (s, 3H), 1.84 – 1.70 (m, 4H), 1.17 (br, 3H), 1.07 (t, J = 7.5 Hz, 3H). LC-MS (ESI, m/z): 652.9[M+H]+.

(**CHMFL-BTK-85B**). To a solution of **S5** (110mg, 0.18mmol) in MeOH was added Pd/C(10%Pd,55%water) (10mg). The reaction mixture was stirred at room temperature for 0.5h, and then resulting mixture filtered through a pad of Celite. The filtrate was concentrated to give a crude *N*-(3-(5-((3-amino-4-(4-ethylpiperazin-1-yl)phenyl)amino)-1-methyl-6-oxo-1,6-dihydropyridin-3-yl)-2-methylphenyl)-4,5,6,7-tetrahydrobenzo[*b*]thiophene-2-carboxamide (80mg). To a solution of the *N*-(3-(5-((3-amino-4-(4-ethylpiperazin-1-yl)phenyl)amino)-1-methyl-6-oxo-1,6-dihydropyridin-3-yl)-2-methylphenyl)-4,5,6,7-tetrahydrobenzo[*b*]thiophene-2-carboxamide (80mg , 0.13mmol) in dry DCM (10ml) was added 4-bromobut-2-enoyl chloride (29mg, 0.16mmol) and DIPEA (34mg, 0.26mmol) at 0 ^o^C. Then the reaction mixture was stirred at room temperature for 0.5h, and then quenched with NaHCO_3_ (aq) and extracted with DCM (3 * 20 mL). The combined organic layers were washed with water, brine and dried over anhydrous Na_2_SO_4_. Evaporation of the solvent provided the crude N-(3-(5-((3-(4-bromobut-2-enamido)-4-(4-ethylpiperazin-1-yl)phenyl)amino)-1-methyl-6-oxo-1,6-dihydropyridin-3-yl)-2-methylphenyl)-4,5,6,7-tetrahydrobenzo[b]thiophene-2-carboxamide (74mg). To a solution of the N-(3-(5-((3-(4-bromobut-2-enamido)-4-(4-ethylpiperazin-1-yl)phenyl)amino)-1-methyl-6-oxo-1,6-dihydropyridin-3-yl)-2-methylphenyl)-4,5,6,7-tetrahydrobenzo[b]thiophene-2-carboxamide (74mg, 0.10mmol) in DMF(5 ml) was added DIPEA (26mg, 0.20mmol) and Amine-PEG3-Biotin (65mg, 0.15mmol). The reaction mixture was stirred at room temperature for 12 h, and then quenched with NaHCO_3_ (aq) and extracted with EA (3 * 20 mL). The combined organic layers were washed with water, brine and dried over anhydrous Na_2_SO_4_. Evaporation of the solvent provided the crude product, which was purified by flash chromatography with0~20% MeOH in DCM to afford **CHMFL-BTK-85B** (11 mg, three steps yield =5.7%) as a red solid. ^1^H NMR (500 MHz, DMSO) δ 9.85 (s, 1H), 9.14 (s, 1H), 8.11 (s, 1H), 7.88 (t, *J* = 4.7 Hz, 1H), 7.71 (s, 2H), 7.30 – 7.11 (m, 5H), 7.01 (d, *J* = 9.1 Hz, 1H), 6.94 (s, 1H), 6.78 (s, 2H), 6.41 (s, 1H), 6.37 (s, 1H), 4.34 – 4.23 (m, 1H), 4.12 (s, 1H), 3.71 (d, *J* = 5.1 Hz, 4H), 3.59 – 3.57 (m, 8H), 3.52 – 3.51 (m, 5H), 3.39 – 3.38 (m, 4H), 3.18 (d, *J* = 5.7 Hz, 3H), 3.05 (s, 5H), 2.96 (s, 5H), 2.81 (dd, *J* = 12.4, 5.0 Hz, 2H), 2.76 (s, 2H), 2.64 – 2.56 (m, 3H), 2.16 (s, 3H), 2.06 (t, *J* = 7.3 Hz, 2H), 1.85 – 1.70 (m, 4H), 1.65 – 1.54 (m, 1H), 1.48 (m, 3H), 1.31 – 1.15 (m, 5H). LC-MS (ESI, m/z): 541.3[M+2H]^2+^.

**Cell lines and cell culture**

The diffuse large B-cell lymphoma (TMD8, WSU-DLCL2, Pfeiffer, SU-DHL-6, U2932, SU-DHL-2), mantle cell lymphoma (REC-1, JEKO-1), follicular lymphoma (DOHH2), burkitt’s lymphoma (Raji, Ramos) were used in the study. TMD8 was provided by Dr. Scott Armstrong, Dana Farber Cancer Institute (DFCI), Boston, MA, USA. WSU-DLCL2, Pfeiffer, SU-DHL-6, U2932, SU-DHL-2, REC-1, JEKO-1, DOHH2, Raji and Ramos were purchased from Cobioer Biosciences CO., Ltd. (Nanjing, China). All cell lines were cultured in RPMI 1640 media (Corning, USA) with 10% fetal bovine serum (FBS) and supplemented with 2% L- glutamine, 1% penicillin/streptomycin and were maintained in culture media at 37 ºC with 5% CO_2_.

**Antibodies**

# The following antibodies were purchased from Cell Signaling Technology (Danvers, MA): Phospho-BTK (Tyr223) Antibody (#5082), BTK (C82B8) Rabbit mAb (#3533), Phospho-PLCγ2 (Tyr1217) Antibody (#3871), PLCγ2 Antibody (#3872), AKT (pan) (C67E7) Rabbit mAb (#4691), Phospho-AKT (Thr473) Rabbit mAb (#4051), p44/42 MAPK (ERK1/2) (137F5) Rabbit mAb (#4695), Phospho-p44/42 MAPK (ERK1/2) (Thr202/Tyr204) (D13.14.4E) XP® Rabbit mAb(#4370), Phospho-NF-κB p65(Ser536) (#3033), NF-κB p65 (C22B4) Rabbit mAb (#4764). Antibodies were used at 1:1000.

**ATP competitive assay**

ADP-Glo^TM^ assay kits from Promega Corporation were used according to instructions. CHMFL-BTK-85 was generally prepared with 1:3 serial dilutions for 4 concentrations (300 nM, 100 nM, 30 nM, and 10 nM). 8 concentrations were used (1 μM to 1 nM) for ATP competition experiments. 2.5 µL BTK (10 ng/µL) was incubated with CHMFL-BTK-85 and CHMFL-BTK-85R for 60 min at room temperature in reaction buffer followed by addition of 2.5 µL ATP/substrate mixture. The assay was conducted for 1 h at 37 °C. After the plate cooled for 5 min at room temperature, 5 μL of ADP-Glo reagent was added into each well to stop the reaction and consume the remaining ADP within 40 min. At the end, 10 µL kinase detection reagent was added and incubated for 30 min at room temperature. The luminescence signal was read with an envision Perkin Elmer plate reader (Envision, PE, USA).

**Cellular BTK phosphorylation**

The effect of CHMFL-BTK-85 and Ibrutinib on cellular wild type BTK and BTK C481S mutant phosphorylation on Y223 was assessed in HEK293T cells transfected with WT BTK or BTK C481S plasmids.

**In vitro target occupation assay**

Cell lysates from REC-1 cells were treated with different concentrations of covalent inhibitor CHMFL-BTK-85 on ice for 2 hours. 1 µM biotinylated covalent probe (CHMFL-BTK-85B) was then added and lysates were incubated for another 2 hours. Uninhibited BTK was then captured by adding 50 µl Streptavidin agarose resin suspensions (Pierce) followed by an incubation for 1 hour. The resin was then spun down, washed briefly with 1X PBS buffer, and then resuspended in protein sample buffer for Western blot analysis.

**Biochemical kinase assay**

The biochemical tests of BMX, BLK, JAK3, TXK, HER2, HER4, ITK, EGFR, and BTK were provided by Invitrogen (Carlsbad, CA, USA).

**Enzyme-Linked Immunosorbent Assay (ELISA)**

Peripheral blood mononuclear cells (PBMC) were separated from the blood of healthy donors by Ficoll density gradient centrifugation (Histopaque-1077, Sigma-Aldrich). Cells were enriched using a MACS® human NK cell negative-selection isolation kit (Miltenyi Biotec, Germany) according to the manufacturer’s instructions. Purified NK cells were cultured with 1000U/ml IL2 for 48h. Enriched NK cells (6 × 10^5^ /ml) were cultured with the same volume of Mino cell and SK-OV-3 cell (1-2 × 10^4^ /ml) for 24 h at 37°C in a humidified CO_2_ incubator, with Rituximab and Herceptin (10 μg/mL), and then with CHMFL-BTK-85 or PCI32765 for 48 hours. The concentrations of interferon (IFN)-γ in cell culture supernatants obtained from three independent experiments were detected using commercial ELISA kits (absin, #abs510007), according to the manufacturer’s instructions. The human IFN-γ kit sensitivity was 7.8 pg/ml.

**Cytotoxicity Assay**

The cytotoxicity of NK cells was measured using the CyQUANT™ LDH Cytotoxicity Assay Kit (Invitrogen™, #C20300) according to the manufacturer’s instructions. Briefly, enriched NK cells (6 × 10^5^ /ml) were cultured with the same volume of Mino cell and SK-OV-3 cell (1-2 × 10^4^ /ml) in a 96-well plate for 4 h with Rituximab and Herceptin (10 μg/mL) and CHMFL-BTK-85 or PCI32765 at 37°C in a humidified CO_2_ incubator prior to removal of the supernatant for lactate dehydrogenase (LDH) release measurements. Calculate % Cytotoxicity by using the following formula: %Cytotoxicity = (Compound-treated LDH activity-Spontaneous LDH activity)/(Maximum LDH activity-Spontaneous LDH activity)*100.

**Proliferation studies**

Cells were grown in 96-well culture plates (2000-4000/well), with various concentrations of CHMFL-BTK-85, Acalabrutinib, and Ibrutinib for 72 h. Cell viability was measured using the CellTiter–Glo assay (Promega, USA) according to the manufacturer’s instructions, and absorbance was measured in a Bio-red Microplate reader (Bio-Red, USA).

**Signaling pathway effect examination**

TMD8, REC-1, and DOHH2 cells were treated with DMSO and serially diluted CHMFL-BTK-85 or Ibrutinib for 4 h, and then stimulated cells with anti-IgM. The cells were then washed in PBS and lysed in cell lysis buffer. BTK, Phospho-BTK(Tyr223), PLCγ2, Phospho-PLCγ2 (Tyr1217), Phospho-AKT Thr473, Phospho-NF-κB p65(Ser536), ERK, Phospho-p44/42MAPK (ERK1/2) (Thr202/Tyr204), AKT and GAPDH antibody (Cell signaling Technology) were used for immunoblotting. For NF-κB p65 western blot, the cell lines were treated with CHMFL-BTK-85 or ibrutinib for 8 hours and then whole-cell extracts were prepared and western blot analysis was conducted for p-NF-κB p65 and GAPDH protein levels.

**Cell cycle analysis**

TMD8, REC-1 and DOHH2 cells were treated with serially diluted CHMFL-BTK-85 or Ibrutinib for 24-48h. The cells were fixed in 70% cold ethanol and incubated at −20 °C overnight then stained with PI/RNase staining buffer (BD Pharmingen). Flow cytometry was performed using a FACS Calibur (BD), and the results were analyzed by ModFit software.

**Apoptosis effect examination**

TMD8, REC-1 and DOHH2 cells were treated with serially diluted CHMFL-BTK-85 or Ibrutinib for 48 h. The cells were then washed in PBS and lysed in cell lysis buffer. PARP, Caspase-3, GAPDH antibody (Cell signaling Technology) were used for immunoblotting.

**In Vivo Pharmacokinetics Studies**

The pharmacokinetics study protocol was approved by the animal ethics committee of Hefei Institutes of Physical Science, Chinese Academy of Sciences (Hefei, China). The male Sprague-Dawley rats were provided by the laboratory animal center of Anhui Medical University (Hefei, China). The animals were housed in an air-conditioned animal room at a temperature of 23 ± 2°C and a relative humidity of 50 ± 10% and allowed free access to tap water and lab. CHMFL-BTK-85 was administered by oral gavage and plasma samples collected 0.033, 0.083, 0.25, 0.5, 1, 2, 4, 6, 9 and 12 hours after dosing. Hundred microliters of plasma was harvested by centrifuging the blood sample at 4 °C and 5000 rpm for 3 min, and then stored at −80 °C until analysis. An aliquot of 100 μL of each plasma sample was mixed with 20 μL of internal standard working solution (200 ng/mL caffeine). Methanol (400 μL) was then added for precipitation. After vortexing for 5 min and centrifuging at 14000 rpm for 10 min, 5 μL of the supernatant was injected for LC−tandem mass spectrometry analysis. Pharmacokinetic parameters were obtained by fitting the normalized liquid chromatography tandem mass spectrometry peak areas to a noncompartmental model using the linear trapezoidal estimation method in the WinNonlin software package (Pharsight Corporation, Mountain View). The oral bioavailability (F) is calculated according to the following equation: F = AUC0−∞ (oral)/AUC0−∞ (i.v.) × dose (i.v.)/dose (oral) × 100%.

**Rat 14-day sub-drug test**

The subacute toxicity study of CHMFL-BTK-85 was conducted on SD rats, 200g in weight and five weeks in age. All mice were purchased from Gempharmatech Co., Ltd (Nanjing, China). All animals were housed in a specific pathogen-free facility and used according to the animal care regulations of Hefei Institutes of Physical Science Chinese Academy of Sciences. CHMFL-BTK-85 was delivered daily in a HKI solution (0.5% Methocellulose/0.4% Tween 80 in ddH_2_O) by orally gavage. A range of doses of CHMFL-BTK-85 or its vehicle were administered, as indicated in figure legends. Body weight was measured daily after CHMFL-BTK-85 treatment.

**TMD8 and REC-1 cell subcutaneous Xenograft tumor model**

Five-week old female SCID mice were purchased from Beijing Vital River Laboratory Animal Technology Co., Ltd (Beijing, China). All animals were housed in a specific pathogen-free facility and used according to the animal care regulations of Hefei Institutes of Physical Science, Chinese Academy of Sciences. Prior to implantation, the cells were harvested during exponential growth. Ten million TMD8 and REC-1 cells in RPMI1640 medium were formulated as a 1:1 mixture with Matrigel (BD Biosciences) and injected into the subcutaneous space on the right flank of SCID mice. Daily oral administration was initiated when tumors had reached a size of 150 to 200 mm^3^. Animals were then randomly divided into treatment groups, each with 5 mice for efficacy studies. CHMFL-BTK-85 was delivered daily in a HKI solution (0.5% Methocellulose/0.4% Tween 80 in ddH_2_O) by orally gavage. A range of doses of CHMFL-BTK-85 or its vehicle were administered, as indicated in figure legends. Body weight and tumor growth were measured daily after CHMFL-BTK-85 treatment. Tumor volumes were calculated as follows: tumor volume (mm^3^) ＝ [(W^2^ × L)/2] in which width (W) is defined as the smaller of the two measurements and length (L) is defined as the larger of the two measurements.

**REC-1 cells bone marrow engrafted mouse model**

Five-week old female NOD-SCID mice were purchased from the Beijing Vital River Laboratory Animal Technology Co., Ltd (Beijing, China). All animals were housed in a specific pathogen free facility and used according to the animal care regulations of Hefei Institutes of Physical Science, Chinese Academy of Sciences. NOD-SCID mice were induced by intraperitoneal injection of cyclophosphamide (CTX) 50 mg/kg daily for two days. 8 million REC-1 cells in 0.3 mL RPMI1640 medium were injected by intravenous after 24 h. CHMFL-BTK-85 or Ibrutinib treatments were initiated daily by *p.o.* 3 weeks after cell inoculation. Mice were monitored daily and were euthanized when moribund or at early signs of hind limb paralysis.

**
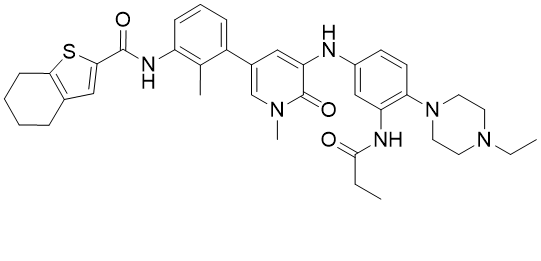
**

**Fig. S1 Chemical structure of CHMFL-BTK-85R**

**
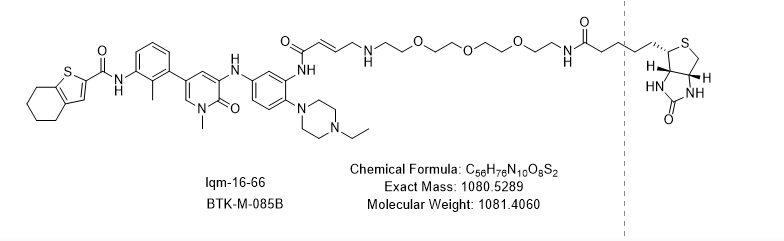
**

**Fig. S2 Chemical structure of CHMFL-BTK-85B**

**
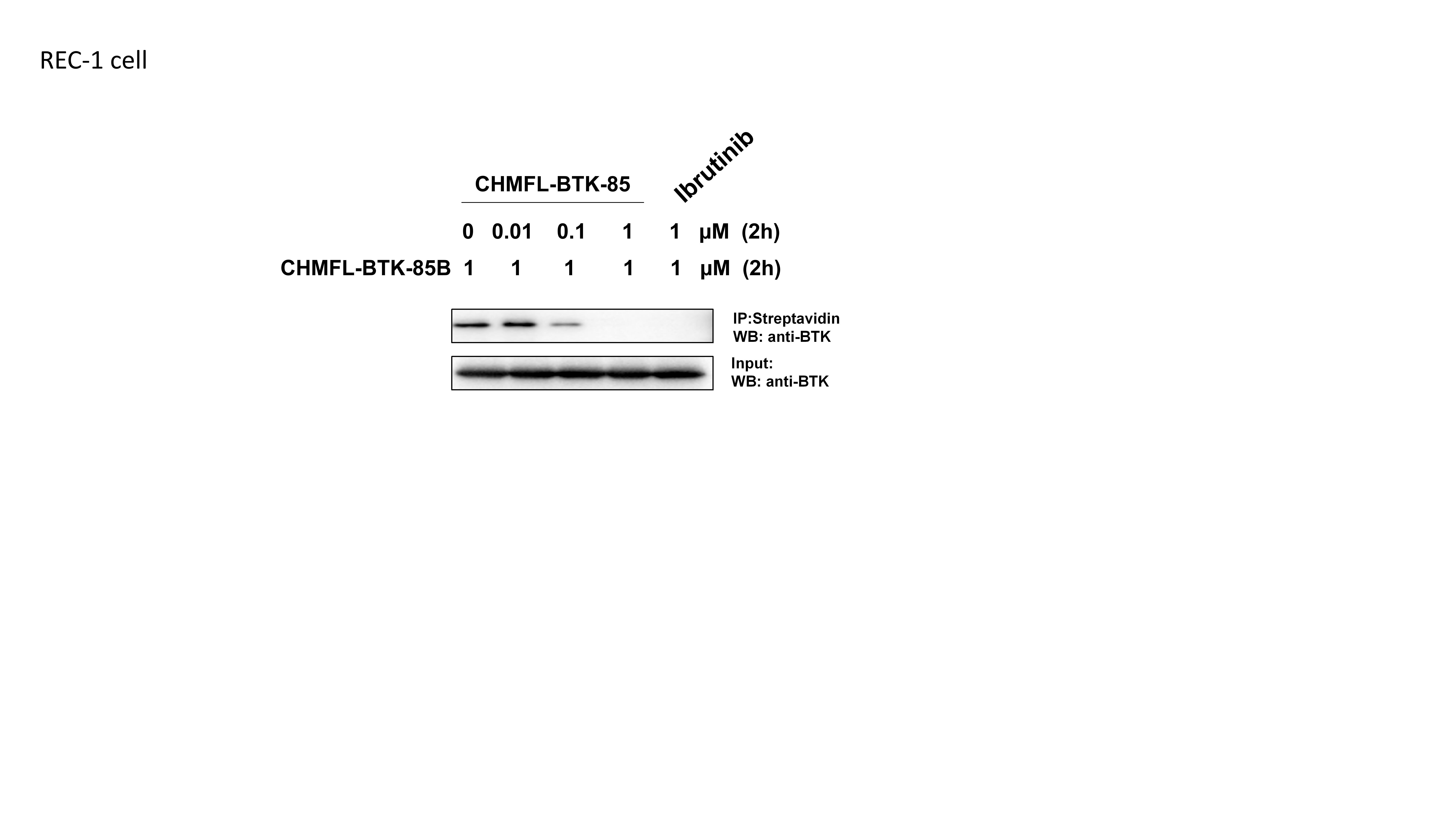
**

**Fig. S3 Target engagement studies using CHMFL-BTK-85 and CHMFL-BTK-85B show that CHMFL-BTK-85 binds BTK in REC-1 cell.**





**Fig. S4 The Interferon (IFN)-γ levels in the conditioned medium were measured by ELISA.** SK-OV3 cells and NK cells were co-seeded and treated with vehicle or various concentrations of BTK inhibitors in the presence of Herceptin for 48h. Date are shown as mean ± SEM, **P*-value < 0.05; ***P*-value < 0.01; ****P*-value < 0.001, and ****p < 0.0001.





**Fig. S5 Cytotoxicity of the target cells was determined by lactate dehydrogenase release into the culture medium.** SK-OV3 cells and NK cells were co-seeded and treated with vehicle or various concentrations of Ibrutinib and CHMFL-BTK-85 in the presence of Herceptin for 4h. Date are shown as mean ± SEM, **P*-value < 0.05; ***P*-value < 0.01; ****P*-value < 0.001, and ****p < 0.0001.

**
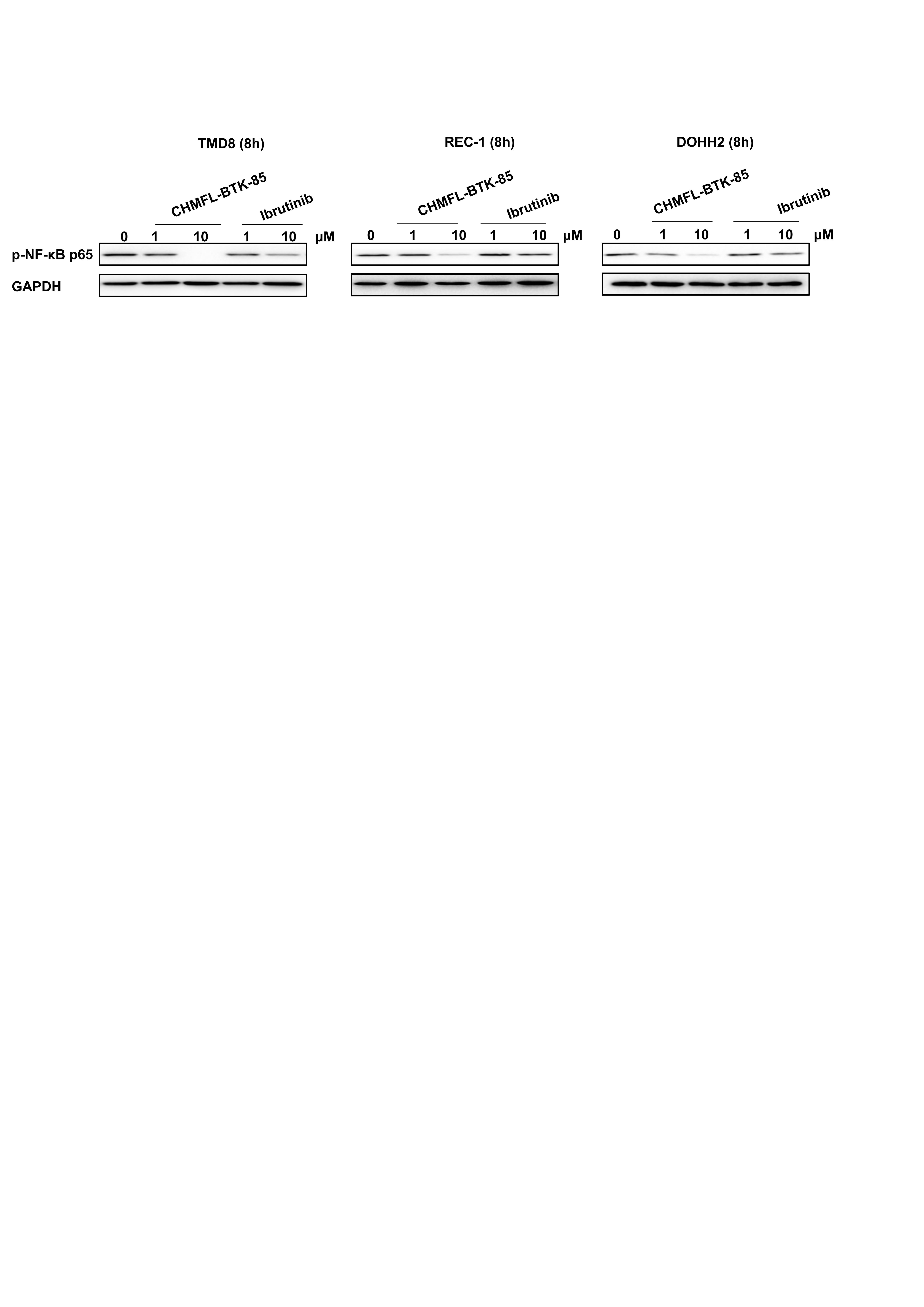
**

**Fig. S6 The phosphorylation levels of NF-κB p65 was detected by western blot in TMD8, REC-1 and DOHH2 cell lines.** These cells were incubated with the indicated concentrations of CHMFL-BTK-85 or Ibrutinib for 8 h before lysis.





**Fig. S7 TMD8, REC-1 and DOHH2 cells were treated with serially diluted** **CHMFL-BTK-85 or Ibrutinib for 24-48 h. a** Flow cytometry was performed using a FACS Calibur, and the results were analyzed by ModFit software. The panels demonstrate quantification of the percentage of cells in each cell cycle phase. **b** Western blot analysis for the expression of PARP, cleaved PARP, caspase-3, and cleaved caspase-3 in TMD8, REC-1 and DOHH2 cells treated with serially diluted CHMFL-BTK-85.

**

**

**Fig. S8 a** Effect of CHMFL-BTK-85 on body weight. **b** The mass of heart, lung, spleen, liver, kidney in the rats at 14 days.





**Fig. S9 a** Body weight monitoring of CHMFL-BTK-85 in TMD8 cell subcutaneous tumor model. **b** Western blot analysis of CHMFL-BTK-85 on BTK signaling pathways in the tumor tissues after 28-day treatment period.

**

**

**Fig. S10** **a** Effect of CHMFL-BTK-85 on body weight. **b** Anti-tumor effects of CHMFL-BTK-85 with once daily (QD) dosing at 50, 100, and 150 mg/kg. The total study length was 14 days. (Left) Representative graphs of relative tumor size are shown. (Right) Representative graphs of tumor weight of different groups are shown (Error bars, mean ± SEM, n = 5. *P-value < 0.05; **P-value < 0.01; ***P-value < 0.001). **c** Body weight monitoring of CHMFL-BTK-85 in REC-1 cell bone marrow engrafted mouse model (left); The median survival time of each treatment group (right).

**Table S1** ADP-GLO^TM^ assay determination of the IC_50S_ of CHMFL-BTK-85 against BTK, BLK, BMX, EGFR, HER2, HER4, ITK, JAK3 and TXK

| **Target** (nM) | BTK | BLK | BMX | EGFR | HER2 | HER4 | ITK | JAK3 | TXK |
| --- | --- | --- | --- | --- | --- | --- | --- | --- | --- |
| **CHMFL-BTK-85** | 11.5±1.6 | >10,000 | 119.8±3.7 | >10,000 | >10,000 | >10,000 | >10,000 | 2723±45 | 3313±17 |

**Table S2 (separate file)**

Kinome wide selectivity profiling of CHMFL-BTK-85 with DiscoveRx KinomeScan^TM^ assay

**Table S3** CHMFL-BTK-85’s anti-proliferative effect against a panel of B-cell lymphoma cell lines

| **Cell** | **CHMFL-BTK-85**  (GI_50_: µM) | **Ibrutinib**  (GI_50_: µM) | **Acalabrutinib**  (GI_50_: µM) |
| --- | --- | --- | --- |
| TMD8 | 0.017±0.004 | 0.001±0.0001 | 0.020±0.003 |
| REC-1 | 0.028±0.007 | 0.03±0.021 | 0.03±0.002 |
| Pfeiffer | 0.29±0.14 | 1.2±0.12 | / |
| SU-DHL-2 | 0.57±0.35 | 4.9±0.2 | / |
| DOHH2 | 0.7±0.15 | 0.26±0.18 | / |
| U2932 | 0.8±0.096 | 1.4±1.2 | / |
| SU-DHL-6 | 0.97±0.22 | 0.37±0.055 | / |
| JEKO-1 | 1.1±0.24 | 2±0.15 | / |
| Ramos | 1.3±0.1 | 3.8±0.66 | / |
| WSU-DLCL2 | 1.4±0.21 | 0.18±0.10 | / |
| Raji | 1.5±0.058 | 6.1±1.8 | / |

**Table S4** Pharmacokinetics properties of CHMFL-BTK-85

| **CHMFL-BTK-85** | **Rats** | |
| --- | --- | --- |
|  | **I.V.** (1mg/kg) | **P.O.** (10mg/kg) |
| AUC_(0‑t)_ (ng/mL·h) | 747±263 | 2145±475 |
| AUC_(0-∞)_ (ng/mL·h) | 766±261 | 2158±474 |
| C_max_ (ng/mL·h) | 1079±390 | 296±67 |
| T_max_ (h) | 0.02±0 | 6±0 |
| T_1/2_(h) | 1.6±0.3 | 2.9±0.1 |
| MRT _(0‑ ∞)_ (h) | 1.6±0.2 | 6.5±0.1 |
| F (%) | / | 29% |
